# The evolutionary biomechanics of locomotor function in giant land animals

**DOI:** 10.1242/jeb.217463

**Published:** 2021-06-08

**Authors:** John R. Hutchinson

**Affiliations:** Structure & Motion Lab, Department of Comparative Biomedical Sciences, The Royal Veterinary College, Hawkshead Lane, North Mymms, Hertfordshire AL9 7TA, UK

**Keywords:** Scaling, Muscle, Gait, Effective mechanical advantage, Maximal speed

## Abstract

Giant land vertebrates have evolved more than 30 times, notably in dinosaurs and mammals. The evolutionary and biomechanical perspectives considered here unify data from extant and extinct species, assessing current theory regarding how the locomotor biomechanics of giants has evolved. In terrestrial tetrapods, isometric and allometric scaling patterns of bones are evident throughout evolutionary history, reflecting general trends and lineage-specific divergences as animals evolve giant size. Added to data on the scaling of other supportive tissues and neuromuscular control, these patterns illuminate how lineages of giant tetrapods each evolved into robust forms adapted to the constraints of gigantism, but with some morphological variation. Insights from scaling of the leverage of limbs and trends in maximal speed reinforce the idea that, beyond 100–300 kg of body mass, tetrapods reduce their locomotor abilities, and eventually may lose entire behaviours such as galloping or even running. Compared with prehistory, extant megafaunas are depauperate in diversity and morphological disparity; therefore, turning to the fossil record can tell us more about the evolutionary biomechanics of giant tetrapods. Interspecific variation and uncertainty about unknown aspects of form and function in living and extinct taxa still render it impossible to use first principles of theoretical biomechanics to tightly bound the limits of gigantism. Yet sauropod dinosaurs demonstrate that >50 tonne masses repeatedly evolved, with body plans quite different from those of mammalian giants. Considering the largest bipedal dinosaurs, and the disparity in locomotor function of modern megafauna, this shows that even in terrestrial giants there is flexibility allowing divergent locomotor specialisations.

## Abstract (precis)

**Summary:** Examining size-related changes in living and extinct taxa reveals that giant land tetrapods become less athletic during their evolution; however, there are multiple evolutionary solutions to the constraints associated with giant size.

## INTRODUCTION

**Fig. 1.**
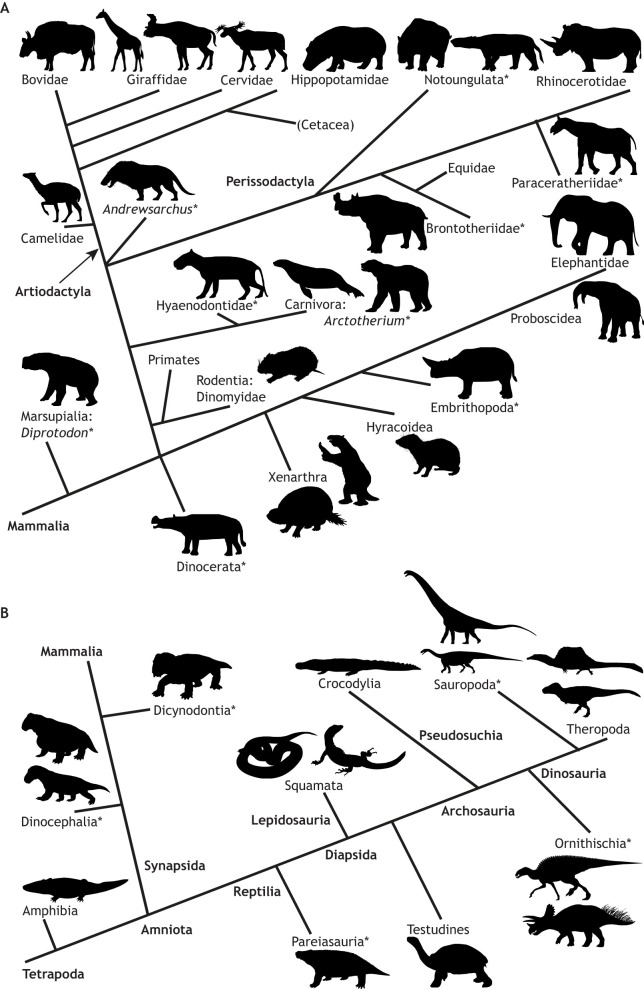
**Phylogenetic relationships of mammals and tetrapods showing giant or near-giant taxa (∼1000+** **kg).** The relationships are shown for (A) mammals and (B) tetrapods. *Extinct taxon. Table S1 details mass estimates and references. Phylogeny from Crawford et al. (2015), Gauthier (1986), Gauthier et al. (1988), Gheerbrant et al. (2018), Halliday et al. (2017), Holbrook and Lapergola (2011), O'Leary et al. (2013), Rowe (1993), Upham et al. (2019), Welker et al. (2015) and Westbury et al. (2017). Silhouettes from http://phylopic.org/, by An Ignorant Atheist, Dmitry Bogdanov, L.-M. Davalos, Tasman Dixon, Ghedoghedo, Scott Hartman, Tracy A. Heath, Jackovche, Chris Jennings, Karkemish, Oscar Sanistro, Roberto Diaz Sibaja, Christopher Silva, Smokeybjb, Nobu Tamura, David Tana, Steven Traver, Jan A. Venter, Mark Witton, Emily Willoughby and Zimices; used under a Creative Commons Attribution 3.0 Unported license (https://creativecommons.org/licenses/by/3.0/) or Public Domain Dedication 1.0 license (http://creativecommons.org/publicdomain/zero/1.0/).

Two patterns prevail when surveying the evolution of legged vertebrates on land (tetrapods) in the context of body size. First, ‘giant’ body size (≥1000 kg body mass; Owen-Smith, 1987) has evolved repeatedly since the Permian period (Alexander, 1998; Vermeij, 2016); initially in non-mammalian synapsids and other Palaeozoic tetrapods – most famously in Mesozoic dinosaurs – and then in mammals shortly after the Cretaceous–Palaeogene mass extinction. Second, the diversity and morphofunctional disparity of gigantic forms today is depauperate compared with just 20,000 years ago (Pleistocene epoch), let alone in the Mesozoic era. Fig. 1 (also Table S1) shows that giant tetrapods have evolved more than 30 times; however, we are left with only five main groups (elephants, rhinoceroses, hippopotamuses, giraffes and bovids) of extant land giants; all herbivorous placental mammals, and all under threat of human-induced extinction (Ripple et al., 2015). Extant Crocodylia barely reach giant size (Table S1) and deserve more study.

**Fig. 2.**
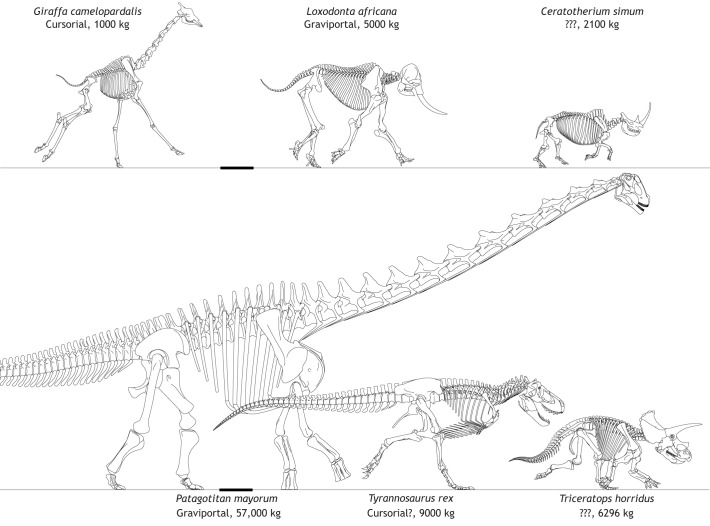
**Morphological specializations of tetrapod limbs across the cursorial–graviportal continuum.** Body masses (kg) are from Table S1. Three extant (upper row) and extinct (lower row) representative giant taxa are shown. ‘???’ emphasizes that a cursorial/graviportal dichotomy does not apply well to these taxa. Scale bars: 1 m. Images by Oliver Demuth.

**Fig. 3.**
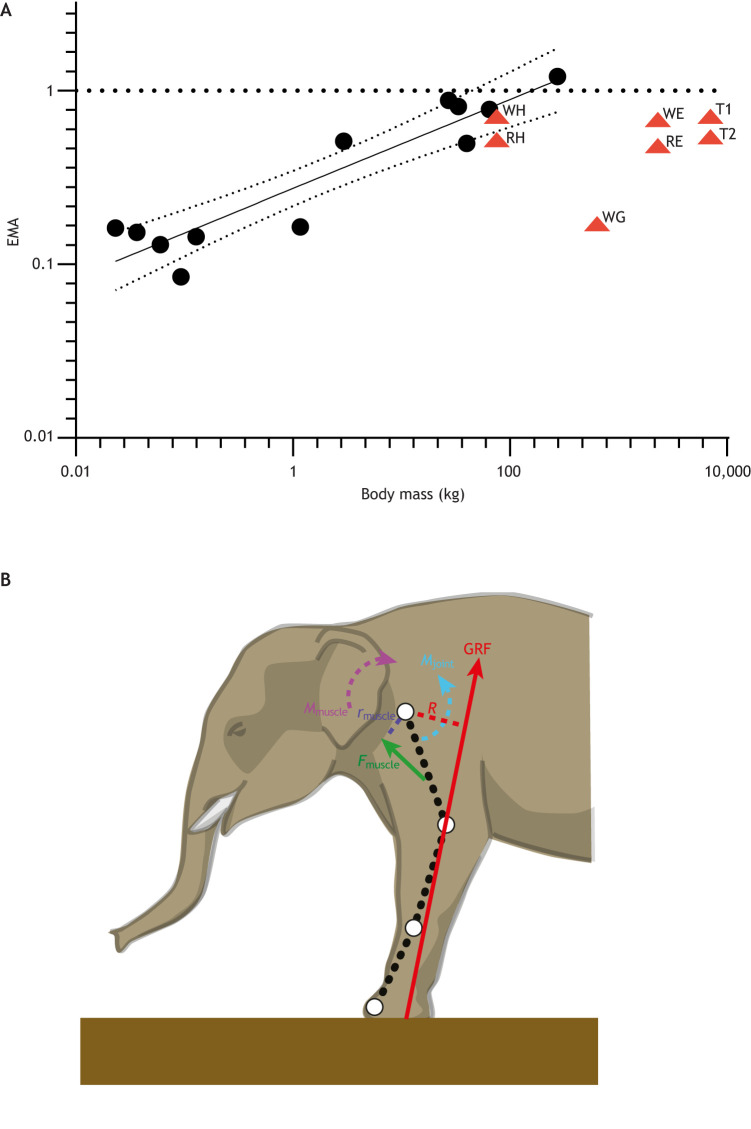
**Effective mechanical advantage (EMA) versus body mass allometry in tetrapods, with an elephant forelimb to explain derivation of EMA.** (A) EMA of most mammals scales as mass^0.26^ up to 274 kg, peaking at 1.2 in horses (Biewener, 2005, black circles). Statistics: linear regression EMA=−0.56Body mass^0.26^ (*N*=12, *R*^2^=0.87, *P*<0.0001; slope 95% confidence intervals=0.19 to 0.33). Data are replotted as a linear regression from Dick and Clemente (2017; from Biewener, 2005). After peaking at 1.2, EMA scaling becomes highly nonlinear, not exceeding 1.0 in any land giants measured to date, but varying widely (∼0.3–0.7), with the addition of the data points shown as red triangles: walking (W) and running (R) humans (WH, RH) (Biewener et al., 2004) and elephants (WE, RE) (Ren et al., 2010); plus two models (T1, T2) ‘bracketing’ potential EMA values of *Tyrannosaurus rex* (Hutchinson, 2004b, with improved input data from Hutchinson et al., 2005, 2011a,b; similar to best limb configurations from fig. 5 in Gatesy et al., 2009); and preliminary data for giraffes (WG) (Basu, 2019; Basu and Hutchinson, 2021 preprint). All data are provided in Table S2. (B) Left forelimb of *Elephas maximus* (adapted from Ren et al., 2010). The ground reaction force (GRF; and internal forces) acting a distance *R* from a joint incur a joint moment *M*_joint_ that must be balanced by an equal and opposite muscle moment *M*_muscle_, produced by muscle forces *F*_muscle_ times their moment arm *r*_muscle_. EMA can be averaged for a limb as *r*/*R* for all joints considered.

Giant tetrapods prompt big questions about the rules of life on land under extreme gravitational constraints. It is timely to unify disparate information from evolution and biomechanics, giving insight into what kinds of giants have evolved and how much diversity in locomotor function could evolve. What special challenges face land giants? How much does giant size force convergence of locomotor form and function during evolution? What are the upper bounds of size and athleticism in nature? A wealth of knowledge has accumulated to answer these questions, but it remains separated in the two disciplinary ‘silos’ of evolution and biomechanics. In this Review, I cover how supportive hard and soft tissues (including control by the nervous system) scale with body mass (i.e. isometrically/allometrically; see Glossary) up to giants, and how giants overcome the challenges of falling versus standing under gravitational constraints (see Glossary). Next, I discuss insights into the biomechanics of giants gained from scaling of limb leverage. I also consider scaling of tissue stresses, safety factors and strength indicators (see Glossary), as well as differential scaling (see Glossary) and its consequences for scaling of maximal speed. In addition, I review our understanding of the locomotor biomechanics of extant and extinct giants, and reflect on how giants evolved through water–land transitions. Finally, using an evolutionary biomechanical perspective, I synthesise our understanding of locomotor function in giant land animals, addressing the questions above to the degree feasible.

Glossary **Aerial phase**

Absence of limb support during a period of locomotion.

**Allometry**

How the size of a particular feature of an organism changes disproportionately in relation to body size (negative, less than isometry; positive, more than isometry).

**Athletic capacity (or athleticism)**

Relative maximal locomotor performance, e.g. maximal speed.

**Constraint**

Limitation on, or canalization of, evolutionary or functional possibilities.

**Cursorial**

Morphology involving elongate distal limb bones; gracile skeleton.

**Differential scaling**

Nonlinear changes with size.

**Duty factor**

Proportion of a stride cycle for which limbs are in stance (support) phase; tending to be inversely correlated with peak GRFs.

**Effective mechanical advantage (EMA)**

Quantitative index of limb leverage (i.e. moment arms *r*/*R*) against gravity; straighter-limbed animals generally have a higher EMA.

**Gait**

Mechanism or pattern of locomotion, e.g. walk or run, trot or gallop.

**Ground reaction force (GRF)**

Force incurred during stance (support) phase of locomotion, equal and opposite to the limb's force and tending to increase with speed.

**Graviportal**

Morphology involving shortened distal limb bones; robust skeleton.

**Inertial delay**

Prolonged response time to perturbations, due to the scaling of muscular capacities to generate accelerations versus body segment inertial properties.

**Isometry**

Scaling with maintenance of proportions to size (i.e. geometric similarity).

**Kinematics**

Motion-related biomechanics (e.g. posture, speed).

**Kinetics**

Force-related biomechanics (e.g. moments, stresses).

**Moment arm**

Leverage of a force (e.g. muscle *r*, GRF *R*) around a joint.

**Safety factor**

Tissue strength/maximal functional stress.

**Strain**

Relative deformation of a structure compared with resting dimensions.

**Strength index**

**Fig. 4.**
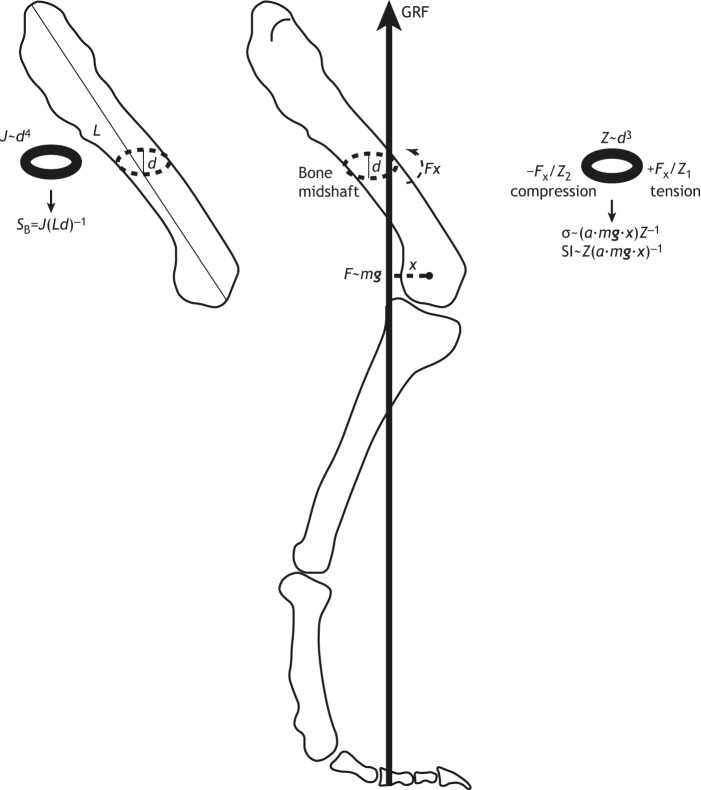
**Strength index and strength indicator derivations for limb bones of land giants.** *Tyrannosaurus rex* right hindlimb shown in side view; redrawn from Gatesy et al. (2009); femur used for example formulae. Strength index *S*_B_ (Selker and Carter, 1989) is derived from the polar moment of inertia *J* (resistance to torsional twisting; related to distribution of bone around the cross-section, i.e. cortical thickness and shape) divided by bone length and diameter (*Ld*). Greater *S*_B_ can result from shorter bones or greater ratios of *J*/*d*. Strength indicator SI (Alexander et al., 1979a,b, 1986; Alexander and Pond, 1992) assumes that a force *F* (proportional to body weight *m**g***, and the GRF) acting with a moment arm *x* around the end of the bone creates a moment *Fx* around the midshaft, used to calculate SI by assuming fraction *a* of body weight (1 in a biped) is borne by the limb and the stress σ resisted by the section modulus *Z* (resistance to bending; related to the ratio of internal versus external diameters of the bone cortices). Greater SI can result from thicker bones (increased *Z*) or reduced load on the bone via decreased *a* or *m**g*** (e.g. GRF) or *x* (e.g. more upright limb posture hence greater EMA; shorter bones can also reduce *x*). *S*_B_ and SI are analogous to safety factors (SI more so because it explicitly estimates bone loads); greater values are ‘safer’ (stronger).

Bone resistance to twisting (*J*) divided by length (*L*) times diameter (*d*), *J*/*Ld* (Selker and Carter, 1989; Fig. 4).

**Strength indicator**

Bone resistance to bending (*Z*) divided by force acting around joint (*a·mg*) times the force's moment arm (*x*), *Z*/(*a*·*mg*·*x*) (e.g. Alexander, 1985a,b; Fig. 4).

**Box 1. Dynamic similarity theory**

Dynamic similarity is an extension of scaling theory (Alexander and Jayes, 1983) used to aid comparisons of animals of different sizes moving at similar relative speeds, and hence to reveal potentially similar relative dynamics (kinetics and kinematics; see Glossary). The scaling of effective mechanical advantage (EMA; Fig. 3) itself is a distortion of this similarity but helps us to understand how some dynamic similarity is maintained with increasing size even if limb orientations are not. The trot–gallop gait transition is often employed as a dynamically similar speed for comparing smaller mammals (in the mouse to horse size range), but does not apply to animals that do not trot and/or gallop, including some modern megafauna (elephants, hippos, giraffes; see ‘Locomotor biomechanics of extant giants’ in main text). Likewise, dynamic similarity tends to assume that geometric similarity (i.e. isometric scaling) applies to the animals being compared, and the distorted limb proportions of giant animals do not comply (Fig. 2). Thus, land giants tend to be an example of ‘breaking’ dynamic similarity via morphological and behavioural changes; their structure and function can no longer be predicted based on trends from smaller animals. Again, methods used to compare smaller animals may only work at small to moderate sizes (mice to horses), adjusting for the confounding effects of size, in the case of dynamic similarity. At best, these methods might only coarsely apply for comparisons with land giants (e.g. Basu et al., 2019a,b). Indeed, some aspects of dynamic similarity are not maintained even across smaller animals – peak joint and other maximal forces scale with strong negative allometry (Alexander, 1980, 1985a,b) – raising the question, how is such scaling explained in terrestrial vertebrates and what does it mean for land giants?

**Fig. 5.**
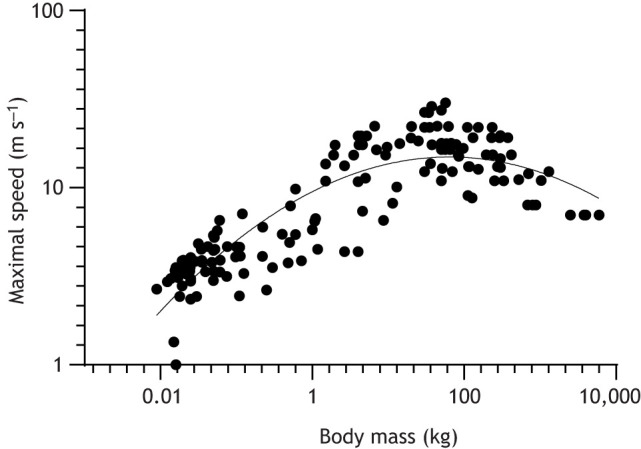
**The relationship between maximal running speed and body mass in mammals is curvilinear.** The graph shows a second-order polynomial fit of data from Dick and Clemente (2017), with modifications noted in Table S3. log_10_Maximal speed=0.9778+0.2170 (log_10_Body mass)−0.06015 (log_10_Body mass)^2^; *N*=150, *R*^2^=0.76, Sy.*x*=0.1617. Larger mammals (>100 kg) cannot reach the top speeds achieved by moderate-sized mammals; indeed, past 1000 kg, few should surpass 10 m s^−1^.

**Box 2. Simple models of locomotion**

The scaling of gait biomechanics other than EMA sheds some additional light on giant tetrapods. Walking and running gaits can be abstracted as variants on a spring-mass or spring-loaded inverted pendulum (SLIP) model, and parameters from experimental studies interpreted in light of that SLIP model and scaling theory. Farley et al. (1993) studied a size range from rats to small horses, inferring or measuring limb mechanics for bouncing gaits via a SLIP model. They found that whole-limb stiffness (force/limb compression) was constant with speed for all species. As speed increased, there was: (1) a flatter trajectory of the centre of mass; (2) a larger arc of limb excursion (but for any speed arcs were constant with size); and (3) greater limb compression (but remaining ∼25% of leg length at a similar speed regardless of size). These changes incurred larger ground reaction forces (GRFs) with speed owing to lower relative ground-contact times. Their findings reinforced the concept of dynamic similarity using the SLIP model, but also prompt questions about the scaling of gait mechanics beyond horses. If EMA is limited in giant tetrapods (e.g. Fig. 3), and thus limb excursions and compression approach some unknown threshold beyond horse sizes, then dynamic similarity would be disrupted for such species, even at similar relative speeds or gaits. This would result from nonlinear scaling of SLIP-type dynamics at larger body sizes. Data from elephants (Ren et al., 2010) hint at such nonlinearity, with EMA <1 and seemingly lower limb stiffness at faster speeds. Follow-up studies elaborated on the above ideas. Herr et al. (2002) found that a SLIP-analogous model of trotting and galloping could obtain similar dynamics to that of Farley et al. (1993) across a similar size range, and using a single control program to maintain stability. Lee et al. (2014) studied a comparable size range with the SLIP model, revealing that goats differ from similarly sized dogs in their more upright posture and greater limb stiffness, thus hinting at EMA divergence between these species (and perhaps Artiodactyla and Carnivora) that is relevant to the scaling of SLIP dynamics.

**Box 3. Can we predict the maximal mass of land giants?**

Hokkanen (1986) – and with lesser success, Economos (1981) – used state-of-the-art understanding of scaling theory (of static bone and muscle strength; or in the latter case ‘gravitational tolerance’ via a very small dataset) to predict the maximal body mass of land animals. The results of their ambitious efforts were that confidence intervals for maximal masses were extremely broad, encompassing both known giants (20,000 kg; Economos, 1981) and masses far beyond those ever measured for land animals (<1,000,000 kg; Hokkanen, 1986). Although today we better understand some basic principles of how land locomotion works (and scales with size), how some giants work and what limits speed, we have no clearer indication on a purely theoretical, mechanistic basis of how big giants could become. We, like Hokkanen (1986) and Economos (1981), remain limited to what we can observe (Fig. 1, Table S1) – 20 tonne mammals and, among dinosaurs, >50 tonne sauropods, <10 tonne bipedal theropods and <20 tonne quadrupedal ornithischians. Additionally, we are limited in that extant land animals (<10 tonnes) or Cenozoic land mammals alone (∼20 tonnes) would give us a biased impression of what is possible. Some land animals became bigger at certain times in the past than now, biasing our current record (due to mass extinctions of giants). Hirt et al. (2017) proposed a biomechanical mechanism explaining the curvilinearity of maximal speed versus body mass. This involved a saturation (in giant land mammals) of the time available to accelerate to maximal speed, although this mechanism did not take into account the differential scaling of force production via EMA scaling (Fig. 3). Their model was approximately similar to those of Meyer-Vernet and Rospars (2015, 2016). Improvements to such a theoretical model (e.g. Fuentes, 2016; Usherwood and Gladman, 2020) might help us to better predict how large giant land animals can become, or how they could move. Encouragingly, Hirt et al.’s (2017) estimates for extinct dinosaurs were roughly concordant with other biomechanical estimates.

### Scaling of supportive tissues

#### Bones

Scaling theory quantifies how animal form and function change with body size, which is valuable for understanding the comparative biology of giant animals. Vast literature on the scaling of vertebrate morphology (such as the limb skeleton), inspired by Galileo's (1638) original insights, considers how body size and form correlate in extant species, and places these findings into biomechanical and behavioural contexts (e.g. Dial et al., 2008). For example, minimal long bone circumference tends to scale isometrically versus body mass – or at most with weak positive allometry – from small tetrapods such as shrews to giant ones such as elephants (Campione and Evans, 2012). However, limb geometry also varies considerably within and across lineages, and within and between bones. The relationship between bone length versus bone diameter or body mass generally shows negative allometry; larger animals tend to have more robust (i.e. shorter, thicker) limb bones, and this is most extreme in giants (Alexander, 1977; Alexander et al., 1979a; Christiansen, 1999; Carrano, 2001; McMahon, 1975a; Prothero and Sereno, 1982; Selker and Carter, 1989). Further variation of limb proportions such as femur/metatarsal length represents the continuum between graviportal and cursorial (see Glossary; Fig. 2) (Carrano, 1999; Coombs, 1978; Gregory, 1912; Osborn, 1929), which becomes conspicuous in giant animals (large bovids and giant giraffes or rhinoceroses versus elephants; or giant theropod versus sauropod dinosaurs; Bertram and Biewener, 1990; Christiansen, 1999; Carrano, 2001; Iriarte-Díaz, 2002; Prothero and Sereno, 1982).

The scaling of vertebrae and intervertebral joints is neglected relative to that of limb bones. Rhinos and large mammals such as bovids have allometrically stiffened thoracic columns with low mobility that is mainly restricted to the lumbosacral joint (Halpert et al., 1987; Jones, 2015; Jones and Holbrook, 2016). Giants such as elephants (and perhaps hippos) seem to have eliminated this mobility, although biomechanical studies of this pattern would be valuable.

Other data on the scaling of bone geometry further illuminate how bone strength is influenced by internal architecture, enabling the support of land giants. Trabecular struts within bones become thicker but more widely spaced in larger land mammals, suggesting that trabecular tissue stress remains constant in joints (Doube et al., 2011). Bone osteons (the primary structural unit of cortical bone) and remodelling capacity follow an analogous trend: osteons and the Haversian canals that run through the centre thereof are reduced in area in larger animals, maintaining overall cortical porosity and thereby strength (Felder et al., 2017). At the level of the whole bone, giant land animals have repeatedly evolved thicker limb bone cortices and filled more of their marrow cavities with cancellous bone. This is sometimes associated with aquatic ancestry (as it confers density and negative buoyancy benefits, facilitating diving; Houssaye et al., 2016a,b). Regardless, these more solid bones should increase skeletal strength; hence, such bones in land giants are either exaptations or adaptations *sensu* Gould and Vrba (1982).

#### Non-skeletal tissue

Scaling of supportive non-skeletal tissues reinforces what the skeletal scaling data reveal – yet the data are more sparse and based on limited sample sizes, and there is underexplored potential for phylogenetic biases. Limb muscles of most mammals, birds and lizards scale with overall allometry of strength-related geometry (>mass^0.67^; Alexander et al., 1979b, 1981; Bennett, 1996; Cieri et al., 2020; Dick and Clemente, 2017; Maloiy et al., 1979; Pollock and Shadwick, 1994a), despite a constant capacity of muscle to produce isometric force per unit area (Close, 1972; Medler, 2002; Rospars and Meyer-Vernet, 2016). In contrast, amniote tendon geometry scales isometrically or with negative allometry, rendering the relatively thinner leg tendons of larger mammals and birds more likely to act in a spring-like fashion; as muscle force/tendon area generally increases with size. This scaling is unaffected by their material properties because these (like those of muscles) remain constant (Alexander et al., 1979b; Pollock and Shadwick, 1994a,b; Bennett, 1996). Articular cartilage thickness in limbs (femoral condyles) of mammals from mice to elephants scales with negative allometry (Malda et al., 2013), consistent with a shift of passive supportive loads from some soft tissues to bones with giant size; but some archosaurian reptiles, including giant sauropods, instead experienced positive allometry (Bonnan et al., 2013; Tsai et al., 2020).

Recent research has revealed the unusual biomechanical problems for the feet of giant land animals. As the locus of interaction between the environment and morphology, feet are major determinants of the biomechanics of locomotion and thus may evolve extreme adaptations in giant tetrapods. Importantly, stress (i.e. foot sole pressure or force/area) increases allometrically in mammals despite greater foot areas (Michilsens et al., 2009; Chi and Louise Roth, 2010; Panagiotopoulou et al., 2012, 2016, 2019; Strickson et al., 2020). Giant mammals thus have repeatedly evolved adaptations to ameliorate increasing foot pressures, such as more upright osteological or functional (‘subunguligrade’) foot postures (Wortman, 1893; Klaits, 1972; Carrano, 1997; Hutchinson et al., 2011a,b; Kubo et al., 2019; Clemente et al., 2020), and foot pads that attenuate impact shocks (Alexander et al., 1986; Warner et al., 2013). Similar features are evident in extinct dinosaurs (Moreno et al., 2007). In megafauna, foot pressures in wild animals seem to be near their limits, and captive animals show increased incidences of mechanically induced pathologies (e.g. osteoarthritis) in regions where pressures are normally high during walking (Panagiotopoulou et al., 2012, 2016, 2019; Regnault et al., 2013, 2017).

The scaling of neuromuscular control is also important for the evolution of giant terrestrial tetrapods. More et al. (2010) and More and Donelan (2018) revealed how the time lags for neural and motor responses to stimuli (e.g. perturbations, reflex arcs and neuromuscular activation) scale with positive allometry in land mammals. Giant tetrapods (e.g. elephants) thereby need more than double the relative response time compared with small animals (e.g. shrews) at fast locomotor speeds, and consequently adopt slower maximal speeds to avoid instability. Inertial delays (see Glossary) are also important for locomotor control. The limb segments of quadrupedal mammals overall scale with negatively allometric resistance to swinging (Kilbourne and Hoffman, 2013, 2015). Mohamed Thangal and Donelan (2020) used biomechanical models combined with the former studies' data on limb inertial properties (and muscular data from Alexander et al., 1981) to estimate the scaling of inertial delays. They found that inertial delays scaled with strong positive allometry (almost tripling in relative magnitude across the shrew-to-elephant spectrum), which meant that, for larger-amplitude motions or in the largest animals, sensorimotor delays may be less influential than inertial delays. This supports the idea that giant land animals should have reduced athletic capacity (see Glossary) to avoid dynamic instability.

#### Standing versus falling challenges for giants

When considering the scaling of supportive tissues, it should be noted that the problem of simply standing may become more important in giants. Large mammalian quadrupeds have more adaptations to standing, such as passive joint-locking mechanisms (Hermanson and McFadden, 1992; Shockey, 2001) and other traits (Weissengruber et al., 2006) that allow grazing or even sleeping while standing still. Perhaps giant tetrapods act more like statically stable systems than smaller taxa (i.e. maintaining their centre of mass over support polygons; Hildebrand, 1980, 1985) rather than relying on more complex dynamic control? Ellis et al. (2018) posited that the large muscular length changes required during standing up from a prone sitting position conflict with locomotor demands, posing a constraint even on moderate-sized tetrapods. Given allometrically shorter muscle fibres, elongate limbs and straighter limb postures in land giants (see Biewener, 2005; Dick and Clemente, 2017), might giants face difficulties in standing up? This question remains unanswered. Falling also presents a greater risk for giant animals, as ‘the bigger you are, the harder you fall’. Whether it is the scaling of sensorimotor control (More et al., 2013; More and Donelan, 2018) or the risk of injury itself (Farlow et al., 1995), land giants seem to have evolved to minimise falling risks. These issues of standing versus falling deserve further neuromechanical research inquiry.

### Scaling of limb leverage

Fig. 3 illustrates the concept of the effective mechanical advantage (EMA; see Glossary) of limbs in tetrapods (Biewener, 1989, 1990, 2005; McMahon, 1975a,b). EMA is the ratio of extensor muscle moment arms *r* (see Glossary; internal; anti-gravity) to moment arms *R* (external; inertially induced) resulting from the ground reaction force (GRF; see Glossary) around joints, which can be averaged for limbs (around mid-stance of locomotion, or as a weighted mean across the stance phase). A higher EMA indicates better overall supportive leverage. Limb postures become straighter as many parasagittally locomoting (i.e. erect; giant taxa in particular) terrestrial tetrapods become larger (Biewener, 1989, 1990, 2005; Osborn, 1900; Reilly et al., 2007). Concurrently, limb muscle moment arms become allometrically larger (Alexander et al., 1981; Biewener, 1990; Maloiy et al., 1979). These changes allometrically increase EMA (Biewener, 2005). However, EMA scaling reaches a plateau around horse-size (perhaps 300 kg in mammals) – big animals cannot become more straight-limbed (limiting minimal *R*) once they are straight-limbed (see below). But as Fig. 3A illustrates, EMA still varies in large land animals – there is not just a simple plateau at EMA ∼1. This variation indicates differential, lineage-specific evolution (i.e. scaling) of EMA in some land giants. Giant tetrapods, then, are not canalized to have a uniform (fully ‘columnar’) limb posture and antigravity support abilities, despite the importance of such abilities in gigantism. Multiple extant giants (e.g. rhinos, hippos) remain unmeasured, and estimates are yet to be made for many extinct taxa; thus, there is likely to be further variation.

EMA (=*r*/*R*) is determined by complex interactions of posture, dynamics and anatomy (Fig. 3B). Musculotendinous moment arms *r* are set mainly by anatomy and somewhat by posture (because moment arms vary with joint angles). The denominator *R* (∼GRF moment arms about joints) is determined largely by posture but also by dynamics (the centre of pressure and GRF vector orientation) and by anatomy (e.g. limb proportions modify where the GRF vector is relative to joints). As above, *R* has its limits in tetrapods, although other relevant anatomical/dynamic parameters such as limb segment masses, centres of mass and inertia can modify it (Biewener et al., 2004). Pike and Alexander (2002) found an interplay between limb proportions and limb kinematics (i.e. posture) in mammalian quadrupeds, implying dependence of EMA on limb proportions, but also perhaps on phylogeny. Hence ‘cursors’ with elongate limb proportions could have low EMA for their size (owing to large *R* caused by long segments; giraffes in Basu, 2019; Basu and Hutchinson, 2021 preprint; tyrannosaurs in Gatesy et al., 2009; elephants in Ren et al., 2010; Fig. 3A). Similarly, there should be trade-offs, with larger moment arms *r* aiding gravitational support but incurring greater musculotendinous excursions (Pandy, 1999), along with larger musculoskeletal anatomy resulting in greater masses or presenting a ‘packing’ problem for fitting tissues onto a body.

Most EMA research on tetrapods has focused on erect, non-sprawling taxa, but results on the scaling of posture and bone in crocodylians and lizards (Blob, 2000; Clemente et al., 2011) suggest that EMA could scale differently in more sprawling tetrapods. Importantly, most previous analyses of EMA have focused on the trot–gallop transition, except for work on walking and running in humans (Biewener et al., 2004) and elephants (Ren et al., 2010). The latter two studies found effects of speed and/or gait (see Glossary) on EMA, so relationships between locomotion and EMA scaling deserve careful investigation, particularly as some giant tetrapods might not trot or gallop and might exhibit postural change with speed and/or gait. It remains unclear, then, how far EMA can be ‘pushed’ in giant tetrapods: an exciting issue for future research. Nonetheless, EMA scaling alone renders giant tetrapods dynamically dissimilar to those in the mouse-to-horse size range (Box 1).

### Stresses, safety factors and strength indicators

Analyses of EMA and spring-loaded inverted pendulum (SLIP) models (Box 2) only roughly indicate how the limbs are loaded, not what the stresses in supportive tissues are. How, then, does the scaling of tissue stress relate to posture and gait? Alexander (1980) estimated that the forces on limb joints across a huge size range (including giant tetrapods) scale inversely with linear dimensions (i.e. as body mass^−0.33^), thereby preserving constant tissue stresses across that size range (Rubin and Lanyon, 1984). Furthermore, ratios of maximal forces exerted on the environment (e.g. GRFs) versus muscle forces are proportional to each other across similar broad size ranges, explaining how peak tissue stresses are maintained within safe bounds (Alexander, 1985a,b). These bounds, effectively ‘safety factors’ or ratios of failure to peak operational stress or strain (see Glossary), concur with results from EMA and bone stress analyses (Biewener, 1989, 1990, 2005; Rubin and Lanyon, 1984). Similarly, Selker and Carter (1989) combined data on internal and external geometry of long bones of artiodactyl mammals (∼15–1500 kg), showing that a ‘bone strength index’ (see Glossary; Fig. 4) scaled in a way that, when combined with likely scaling of muscle forces, would maintain constant peak bone stress. Analogous concepts of safety factors have been used in a ‘strength indicator’ by Alexander et al. (1979b) and others (Fig. 4; see below).

However, we should be cautious in assuming uniform scaling of safety factors. Variation in their magnitudes appears wider than the commonly cited 2–4 ‘constant’, partly owing to the likelihood that true peak stresses and strains have not been measured or estimated for most species (e.g. because of difficulties in obtaining maximum speeds during experiments). For example, Alexander et al. (1979b) assumed hindlimb peak vertical GRFs of 57% body weight and that their elephant subject was near maximal speed, but more precise measurements from Ren et al. (2010) indicate that ∼100% body weight is more accurate, leading to a safety factor of ∼1 (not 2) for muscles and bones. Furthermore, Nunamaker et al. (1990) measured racehorse metacarpal bone strains approaching safety factors of 1 at near-maximal speeds, versus >2 measured at moderate speeds (e.g. Rubin and Lanyon, 1984). Alexander (1997) noted that safety factors are likely to be optimised for the most valuable or vulnerable elements in a ‘mixed chain’ such as a limb, as these elements could be most costly in terms of survival and natural selection. Distal elements may have lower safety factors, perhaps owing to trade-offs in terms of the cost required to swing more robust, stronger but heavier elements (Currey, 2002; Nunamaker et al., 1990). This possibility has been little explored in the context of locomotor scaling (but see Blob et al., 2014) and contrasts strongly with the concept of symmorphosis (evenly matched safety factors across ‘chains’) that much scaling literature implies (e.g. Rubin and Lanyon, 1984). As noted above on the positive allometry of foot sole pressures in mammals, such proximo-distal decreases in safety factors are especially relevant to giant tetrapods.

### Differential scaling and its consequences for scaling of maximal speed

A ‘differential scaling problem’ arises from the above scaling patterns of supportive tissues, leverages and dynamics combined. Consequently, giant tetrapods become ‘nonlinearly constrained’ in their locomotor abilities: elephants and rhinoceroses are not biomechanically giant mice – one cannot simply scale smaller animals up to giant tetrapods because the constraints imposed on them become excessive past moderately large sizes. Much of the gait dynamics scaling literature only considers the realm of mouse-to-horse scaling (e.g. Heglund et al., 1974; SLIP models in Box 2) – dynamic similarity (Box 1) tends to ‘break’ beyond this range, although it depends on the criteria that are considered. For example, giant tetrapods benefit from greater migration distances, but migrate a similar number of body lengths as smaller tetrapods (Hein et al., 2012), preserving some approximation of dynamic similarity; however, larger tetrapods choose slower preferred walking speeds (Lees et al., 2016), acting in contradiction to strict dynamic similarity. Deviations from near-constant stresses or safety factors as outlined above would constitute more contradictions. Another dynamically dissimilar scaling pattern is the nonlinear reduction of maximal speed capacity past moderate sizes, as follows.

Although accurate measurements of maximal speed are infamously scarce, estimates of these speeds were used in a highly influential scaling study by Garland (1983), revisited and corroborated by subsequent papers (Garland and Janis, 1993; Christiansen, 2002; Iriarte-Díaz, 2002; Li et al., 2011; Meyer-Vernet and Rospars, 2015, 2016; Fuentes, 2016; Hirt et al., 2017; Dick and Clemente, 2017; Usherwood and Gladman, 2020; also see Bakker, 1971). At >100 kg body mass, (absolute) maximal speed plateaus, then declines at greater sizes (Fig. 5); the relationship between body mass and maximal running speed is curvilinear, not linear. Hill's (1950) theoretical proposition that maximal speed is mass-independent (which would imply maintenance of dynamic similarity) is thus incorrect, except for a narrower size range (∼10–100 kg) of mammals, including within clades [e.g. Artiodactyla (excluding *Hippopotamus*), Carnivora, Rodentia; Alexander et al., 1977; Garland, 1983]. No ≥1000 kg extant terrestrial mammal is capable of horse-like speeds (Table S2). A common question in the maximal speed scaling literature is how well limb morphology (e.g. ‘cursoriality’) predicts locomotor performance such as maximal speed, or, in another sense, whether morphology is optimised for sprinting speed. The general consensus (*contra* Bakker, 1971) is that morphology instead optimises the metabolic cost of transport at slower ‘cruising’ speeds, whereas maximal speed capacity has multiple morphological ‘solutions’ (Fig. 5), including in giants (Garland, 1983; Garland and Janis, 1983; Carrano, 1999; Iriarte-Díaz, 2002; Christiansen, 2002).

Arguably though, little has changed for our understanding of speed–mass scaling since Garland's classic study, except better measurements of speed in some taxa. Of particular importance for this Review, elephants *Loxodonta africana* and *Elephas maximus* (6000 and 4000 kg, respectively) move at similar maximal speeds ∼7 m s^−1^ (Hutchinson et al., 2003, 2006; also maximal field-based measurements of 5.27 m s^−1^ by Ngene et al., 2010). Speeds of ∼9.7 m s^−1^ for *Loxodonta* (Garland, 1983; Hirt et al., 2017; Table S1) remain speculations. Furthermore, some other maximal speed estimates used for giant mammals seem excessive (e.g. 16.7 m s^−1^ for 1000 kg giraffes versus ≤11 m s^−1^ in Alexander et al., 1977; Basu et al., 2019a). Regardless, such amendments would only strengthen the conclusion that maximal speed declines steeply with mass in giant land mammals. Relative maximal speed (size-normalised via dynamic similarity; Box 1) also declines steeply with giant size in land animals (Alexander et al., 1977; Alexander and Jayes, 1983; Iriarte-Díaz, 2002; Meyer-Vernet and Rospars, 2015, 2016; Usherwood and Gladman, 2020). As a result, whole gaits can be lost at giant size. For example, elephants do not use gaits with aerial phases (see Glossary; Hutchinson et al., 2003).

### Locomotor biomechanics of extant giants

Given the various aspects of scaling discussed above, what do we know about the locomotor biomechanics of existing land giants? Do these giants make use of similar biomechanical solutions to the challenges of locomotion? Large (∼300–1000 kg) tetrapods raise the question of what a ‘giant’ is; they lie in a ‘grey area’. Horses are a prime example – they push the limits of athleticism at large size, combining cursorial anatomy with high EMA to achieve rapid speeds. Bovid artiodactyls likewise are good examples of extreme performers at large or even giant size (some gaurs and other buffalo/bison). This is probably partly achieved via allometric scaling of morphology (McMahon, 1975a,b; Alexander et al., 1977, 1979a,b). A study of EMA scaling up to giant artiodactyls could give valuable insights, following up on Pike and Alexander's (2002) kinematic data for this group. Alexander (1991a) cautioned, however, that, for their size, horses and bovids are neither faster nor more energetically efficient than other mammals, such as carnivores. Some horse-sized mammals can jump and gallop, but some cannot; reinforcing that >300 kg tetrapods lie near thresholds of ‘functional gigantism’ where constraints can reduce athleticism (Biewener, 1989, 1990, 2005; Rubin and Lanyon, 1984).

With only extant mammals to judge from directly, we should be wary of phylogenetic biases. Morphology alone does not reveal which giants are the most athletic, as multiple morphologies can have similar functional outcomes (Wainwright, 2005). Only with biomechanical analyses, testing the role that morphological traits play, can we tease apart where and why specific taxa fall along this spectrum of size versus performance. However, there are too few examples of such analyses of megafauna. What do the locomotor biomechanics of elephants, rhinos, hippos and giraffes (the four other main clades of extant mammalian megafauna) tell us about land giants in general?

Elephants are the most studied land giants in biomechanical terms (Marey and Pagès, 1887; Gambaryan, 1974; Hildebrand, 1984; Hildebrand and Hurley, 1985; Alexander et al., 1979b; Hutchinson et al., 2003, 2006; Fischer and Blickhan, 2006; Ren et al., 2008, 2010; Genin et al., 2010). Although elephants use a columnar limb posture during walking, they gradually shift to a more flexed limb orientation as their speed increases, concurrent with increasingly more bouncing mechanics that indicate a subtle gait shift into biomechanical running (spring-mass mechanics; Box 2). Elephants' EMA never approaches that of a horse at ∼1, instead being remarkably similar to that of walking and running humans at 0.7 and 0.5, respectively (Fig. 3, Table S3) (Biewener et al., 2004; Ren et al., 2010). This postural shift with speed and gait fits the interpretation that elephants trade off the ability to surpass a merely walking gait (despite never approaching an aerial phase and a classical ‘run’) and the maintenance of safe tissue stresses by reducing their maximal speed to ∼7 m s^−1^. The reduction of EMA with speed in elephants should elevate tissue stresses concurrent with increased GRFs, so faster speeds or gaits should become dangerous. Weissengruber et al. (2006) showed how the small menisci of elephant knees correlate with concave proximal cotyles of the tibia for articulation with the femoral condyles, producing a highly congruent knee joint articulation that, with a four-bar linkage mechanism of ligaments, ensures a stable knee throughout the oblique ‘screw-home’ flexion of the joint, particularly in a columnar pose. This anatomy, furthermore, provides a ‘search image’ for similar graviportal specializations in extinct forms.

The biomechanics of (white) rhinoceroses (*Ceratotherium simum*) have only once been studied (Alexander and Pond, 1992). This study showed that peak limb bone stresses, estimated for a galloping gait of ∼7.5 m s^−1^, were about one-third the values estimated for elephants (Alexander et al., 1979b). This fits with the explanation that the shorter, more robust bones in rhinos confer a higher ‘strength indicator’ (Fig. 4) versus elephants (see also Christiansen and Paul, 2001). Clearly, rhinos are more athletic than elephants. They are able to gallop with an aerial phase at speeds faster than an elephant (Gambaryan, 1974; Dagg, 1973; Garland, 1983). Yet it is unclear whether bone strength can explain why rhinos are so athletic even at ∼3000 kg, or whether bone strength is a side effect of other adaptations that are more closely linked to maximal speed capacity, such as muscle or tendon strength. Intriguingly, Prothero and Sereno (1982) found dramatic positive allometry of long bone diameter versus length in rhinos and their relatives. Hence, compared with elephants (Fig. 2), rhinoceroses have much more compact, robust proximal limb bones (femur and humerus). Bakker (1971), Christiansen and Paul (2001) and Paul and Christiansen (2000) added that it is the ‘flexed’ limb posture of rhinos that confers their speed, as compared with the ‘columnar’ posture of elephants (following the morphological logic of Osborn, 1900; for a new morphometric perspective, see Mallet et al., 2019, 2020; also Etienne et al., 2021). There is little question that the limb posture of rhinos and elephants at top (or any) speed is different, but there is much left to be understood about the locomotion of rhinos. Christiansen and Paul (2001) found that long bone strength indicators declined with size from ∼40–6000 kg mammals, with values for elephants (7–13) and *Ceratotherium* rhinos (12–24) slightly overlapping, being greater in general for proximal elements (as per the ‘mixed chain’ hypothesis discussed above). They cautioned that the rhino from Alexander and Pond (1992) was juvenile, and their data indicated that juveniles have higher strength indicators than adults.

No detailed, land-based biomechanical studies have been performed on either giant common hippos (*Hippopotamus amphibius*) or their pygmy cousins (*Choeropsis liberiensis*). Even at ∼3000 kg, large hippos can trot but do not gallop (Dagg, 1973; Hildebrand, 1980), and they have limited speeds of ∼7 m s^−1^ (Garland, 1983). Limb bone strength indicator estimates from Christiansen and Paul (2001) are interesting because they are so low, at even less than elephantine values for a 2400 kg hippo (range ∼6–9). Coughlin and Fish (2009) made the important observation that hippos ‘punt’ underwater using an asymmetrical cantering/galloping gait (*contra* Dagg, 1973), unlike their terrestrial locomotion. Relaxation from the biomechanical constraints of gravity, then, might allow large hippos to express a motor control pattern that is otherwise suppressed; raising the question of whether dwarf hippos ever express that gait on land, or whether hippos in general are constrained anatomically or phylogenetically (e.g. by having large-bodied ancestors with stiffened vertebral joints).

Giraffes, like large bovids, straddle the boundary between large and giant megafauna, and their locomotor abilities reflect that status. Their locomotor kinematics and kinematics generally maintain some dynamic similarity (Box 1) with smaller quadrupeds, except that they adopt lower stride frequencies, related to their apomorphically elongate limbs (Basu et al., 2019a). Those long ‘cursorial’ limbs might incur penalties to EMA (Basu, 2019; Basu and Hutchinson, 2021 preprint) and further trade-offs with maximal performance, since giraffes are slower than horse-sized mammals (maximal speed ∼11 m s^−1^; Dagg and Vos, 1968; Alexander et al., 1977; Basu et al., 2019a). Sensorimotor responsiveness is also slower due to the elongate limbs (More et al., 2013). Although large giraffes can still gallop (but do not trot; Dagg, 1973; Dagg and Vos, 1968), they do so more sedately than smaller individuals, shifting limb kinematics in ways that reduce peak forces and enable this athleticism (Basu et al., 2019a,b).

Thus, from the above points, it is evident that modern mammalian megafauna maintain some locomotor diversity despite their giant sizes. A large rhinoceros seems able to move faster than a similarly sized elephant (or hippo), probably because it is built differently and adopts a different posture, giving it biomechanical benefits that remain obscure (Alexander et al., 1979b; Alexander and Pond, 1992; Christiansen and Paul, 2001; Paul and Christiansen, 2000; Ren et al., 2010; Mallet et al., 2019, 2020). From mice to giraffes, maximal (trot–gallop) stride frequency (strides s^−1^) scales as ∼body mass^−0.14^ (Heglund et al., 1974; Alexander and Maloiy, 1984). Elephants fit this trend surprisingly well: a 2790 kg adult Asian elephant ‘ambling’ at 6.8 m s^−1^ has a stride frequency of 1.6 Hz (Hutchinson et al., 2006), 101% of the predicted value, similar to a ∼750 kg white rhinoceros galloping at 1.7 Hz (Alexander and Jayes, 1983; Alexander and Pond, 1992), 91% of that predicted. However, a ∼1000 kg giraffe galloping at 11 m s^−1^ (Alexander et al., 1977) has only 66% of the predicted stride frequency at 1.2 Hz, because it takes much longer strides (∼9.2 m versus 4.4 m in the other two giants). Consequently, (smaller) giraffes can obtain lower duty factors (see Glossary) (∼0.23) and greater peak GRFs when compared with larger giants (duty factors are 0.48 and 0.39 for the elephant and rhino, respectively). These differences are consistent with the conclusion (from data above and in Fig. 5) that stride length may scale with negative, differential allometry at giant sizes; e.g. the rhino has 100% but the elephant only has 62% of mass-predicted stride lengths (Heglund et al., 1974). This stride length reduction correlates with reduced limb excursion arcs (Box 2), and more upright limbs owing to increases in EMA (Fig. 3), and underpins the reduced athleticism of land giants, including smaller maximal GRFs (Alexander, 1985a,b).

Yet do only four mammalian lineages really teach us all there is to know about locomotor diversity in land giants? Just <20,000 years ago there was a greater diversity of megafauna; before that, dinosaurs achieved ∼170 million years of gigantism. What, then, can the fossil record teach us about the evolutionary biomechanics of gigantism?

### Locomotor biomechanics of extinct giants

What are the main groups of extinct giants and when did they exist? Fig. 1 and Table S1 emphasise that although no amphibians reached giant sizes even while amphibious in habit, there were Permian or later taxa in both the synapsid and reptilian lineages that may have reached 1000 kg in mass, or at least surpassed 500 kg. Cretaceous or later Crocodylomorpha/Crocodylia repeatedly evolved giant sizes even while maintaining some degree of terrestriality. Dinosaurian giants are covered below. The biomechanics of the several giant (or large) Permian/Triassic synapsids remain almost unstudied, although judging from their graviportal morphology, they were relatively slow.

As for mammals, at least one giant marsupial (*Diprotodon*) existed until the late Pleistocene – a graviportal and slow scaled-up version of its wombat kin (Wroe et al., 2004; Price, 2008). Similar robust, graviportal placental mammals evolved soon after the extinction of non-avian dinosaurs, within the Palaeocene/Eocene, including horned Dinocerata and Embrithopoda (followed by Brontotheriidae), carnivorous Hyaenodontidae and mysterious *Andrewsarchus* (known from an enormous skull), and diverse forms of Notoungulata, Litopterna and proboscidean-like Astrapotheria. There were also radiations of still-extant (but now mostly smaller-sized) lineages, such as Xenarthra (giant ground sloths and armadillos), Proboscidea, rhinoceros-kin (Paraceratheriidae, long-legged and long-necked giants), at least one giant equid (*Equus giganteus*) and giant Carnivora such as *Arctotherium* bears. Among Artiodactyla, there were giant short-necked giraffids (*Sivatherium*; Basu et al., 2016), even larger hippos (*Hippopotamus gorgops*), various large or giant bovids (*Bison latifrons*; *Pelorovis*), and giant camelids and cervids (Table S1). Few of these lineages have been studied biomechanically, at best having body mass estimates or bone scaling/strength indicator data incorporated into comparative analyses. However, none seem to have exhibited extreme locomotor adaptations (e.g. high EMA or fast speed/gait capacity). In contrast, giant dinosaurs – in part because they feature the largest bipedal and quadrupedal land animals ever – are well studied from an evolutionary biomechanics perspective. However, a review of dinosaur biomechanics is beyond the scope of this paper (see Hutchinson, 2006 and Alexander, 2006).

The original application of locomotor biomechanics to dinosaurs, or other extinct giants, in a modern sense is best attributed to Alexander (1985a,b, 1989, 1991b). He used simple static models to estimate body mass, centre of mass and thereby bone strength indicators (Fig. 4), with comparisons to similar estimates for extant animals, to gauge the athletic abilities of extinct forms. On this basis, he inferred that giant sauropods (>10 tonnes) should have been no more athletic than elephants; the >6 tonne bipedal theropod *Tyrannosaurus* was about as fast as elephants and sauropods, but the largest ceratopsids such as *Triceratops* (elephant-sized at >6 tonnes) might have been as athletic as rhinos. Alexander (1985a,b) also estimated that foot pressures of sauropods were higher than in extant mammals, consistent with allometric scaling (Michilsens et al., 2009). Farlow et al. (1995) replicated a bone strength indicator for *Tyrannosaurus* similar to Alexander (1985a,b) with better data, but argued that falling risk might limit speed more than bone strength. Christiansen and Paul (2001) and Paul and Christiansen (2000) focused on ceratopsian dinosaurs, finding allometric scaling for long bones comparable to that of (large) mammals and, combined with arguably higher safety factors as in rhinos versus elephants, they argued that even giant ceratopsids might have been as athletic as large rhinos (e.g. able to gallop). These inferences already hinted at remarkable locomotor diversity in just three dinosaur groups, although the claims depend on, among other potential weaknesses noted by Alexander (1991b), how useful bone strength indicators are. These safety factor-like parameters assume that bone strength is the key limiting factor on locomotor performance, not other tissues or stability (see More et al., 2010, 2013; More and Donelan, 2018). Later studies questioned those claims.

Hutchinson and Garcia (2002), Hutchinson (2004a,b) and Gatesy et al. (2009) used simple two-dimensional static models of bipedal dinosaurs to test how well hindlimb muscles could support the moments (rotational forces) involved in fast running and which limb postures were feasible at near-maximal speeds. These analyses drew on emerging biomechanical evidence that muscular force production limits maximal speed. Muscular force production determines limb forces, thereby incurring an equal and opposite GRF (Figs 3B, 4), which is inversely related to ground contact time or duty factor. By applying these methods to extant taxa for validation, they found that giant theropods such as *Tyrannosaurus* were unlikely to have been able to run quickly (≥11 m s^−1^), but might have been able to achieve slow running using certain upright limb poses (Gatesy et al., 2009).

Sellers and Manning (2007) used forward dynamic predictive simulations for some of the same models of *Tyrannosaurus* and obtained similar results, predicting maximal speeds of <9 m s^−1^, supported by follow-up studies (Bates et al., 2010, 2012) for other giant theropods. Further analyses of the various modelling assumptions used – such as muscle moment arms (Hutchinson et al., 2005) and centre of mass (Hutchinson et al., 2007, 2011a,b; Bates et al., 2009) – added reassurance that the fundamental inferences were sound. Sellers et al. (2013, 2017) conducted the most sophisticated simulations yet, for a reconstruction of the ∼80 tonne sauropod *Argentinosaurus* and a 7200 kg model of *Tyrannosaurus*. These produced slow (∼2 m s^−1^) muscle-driven walking estimates for the former taxon, whereas results for the latter suggested that bone strength, rather than muscle strength, limited speed to <8 m s^−1^. There are lingering concerns about the above biomechanical approaches: all contained some unknown inputs (e.g. muscle properties; body segment dimensions), and it is not clear whether static models are sufficient representations of dynamic sprinting, whether predictive simulations are sufficiently valid, or whether assumptions particular to some approaches are reliable. But clearly this domain of modelling research has led the way since the 1980s for studying the evolutionary biomechanics of extinct giants (e.g. Bishop et al., 2018).

As Alexander (1991b) warned, there are pitfalls in analyses of the biomechanics of giant extinct tetrapods, but the stakes are high. Extinct taxa offer immense potential to complement what extant taxa tell us about the biomechanics of land giants, allowing us to build broader biomechanical theory and knowledge. We need extinct animals for a full understanding of how giant size influences terrestrial locomotion; they expand our sample size via their phylogenetic diversity and morphofunctional disparity. Experimental biologists may not recognize this huge opportunity, but palaeontologists and some theoretical biomechanists long have (Bakker, 1971; Alexander, 1985a,b, 1989; Carrano, 2001; Christiansen and Paul, 2001). For example, Carrano's (2001) large dataset on limb bone dimensions in extant and extinct mammals and dinosaurs shows common patterns of negatively allometric scaling of length versus diameter, strongest for larger taxa, consistent with differential scaling. These patterns reinforce the idea that similar biomechanical constraints have faced giant tetrapods from the Jurassic to the present, causing repeated convergent evolution to similar bone allometry and changes of limb posture and athleticism. Thus, palaeontological evidence heavily bolsters insights gained from depauperate extant megafaunas.

### Water–land transitions, gravity and giants

Together, the neontological and palaeontological evidence discussed in the previous two sections reveals that gravity is a biomechanical constraint that results in specializations in giant tetrapods that are not normally evident in smaller taxa. The few cases where tetrapods have made water–land evolutionary transitions involved major shifts in gravitational constraints on locomotor biomechanics – can the study of these transitions therefore inform us about biomechanical adaptations necessary for the evolution of terrestrial giants from smaller terrestrial animals? Arguably the greatest water–land transition was in the ‘rise to land’ of the first tetrapods (Clack, 2012), exapting their limbs from less gravitationally constrained hydrodynamic roles (e.g. bottom-walking) to terrestrial walking and eventually running (Pierce et al., 2012; Reilly et al., 2006). One could view this transition as equally challenging as the later evolution of land giants from smaller ancestors. Giant elephant seals maintain moderate athletic performance (<3 m s^−1^) on land despite their size by relying on axial undulation (Tennett et al., 2018). Another fascinating, and still mysterious, shift is that of ancestral, smaller proboscideans to suddenly large and gigantic forms and their extant elephantid descendants. Palaeocene/Eocene Proboscidea were still largely amphibious, retaining the aquatic habits, plantigrade foot posture and perhaps more sprawling limb posture of their tethythere ancestors (Court, 1993, 1994), but as their body size increased in the Eocene, their limb dimensions became more like those of extant elephants, as did their foot morphology (Hutchinson et al., 2011a,b). Elephants retain some aquatic proficiency and other traits revealing that distant ancestry (Gaeth et al., 1999). This rare water–land transition is in contrast to the common land–water reversals in other tetrapod lineages, in which releases from the gravitational constraints of land allowed radiation into giant sizes (Heim et al., 2015; McClain et al., 2015). This makes Proboscidea a fascinating case study and might help to explain why some of their current locomotor adaptations are so different from those of other giants.

### Synthesis: evolutionary biomechanics of giant tetrapods

A stronger synthesis is needed to explain how land giants deal with the challenges noted above. How much convergence or divergence has there been in the evolutionary history of giant tetrapods on land, and how many ‘solutions’ are there to the constraints and challenges involved? Available evidence suggests some trends for allometry of tissues and control systems, as well as straightened limb posture and/or increased EMA and decreased locomotor performance with increasing size. However, there is also persistent variation at giant sizes in extant and extinct lineages, perhaps explaining why it seems impossible to predict how large giant tetrapods can become (Box 3). That variation is analogous to patterns described by Dick and Clemente (2017) for felid mammals and varanid lizards, two groups with broad size ranges. Felids are less crouched (have an apparently higher EMA) than varanid lizards, which maintain safe tissue stresses via allometrically larger muscles (Dick and Clemente, 2016; Cieri et al., 2020). Felids, then, may simply cope with being weaker at larger sizes and reducing relative athletic capacity more steeply with increases in size, but neither felids nor varanids abandon whole gaits as their size increases. It would be exciting to know, then, what the maximal performance of the giant varanid *Varanus priscus* was; how far did it reduce its athleticism compared with the extant komodo dragon *V. komodoensis* (<1000 versus <300 kg; Fry et al., 2009)?

Future studies should aim to understand land giants in relative, not just absolute, terms. Both ontogenetic (intraspecific) and evolutionary (interspecific) scaling involve allometrically reduced maximal relative performance (Carrier, 1983; Clemente et al., 2009, 2012; Herrel and Gibb, 2006; Pennycuick, 1975; Smith and Wilson, 2013). Which lineages lose discrete modes of locomotion during ontogeny/growth? Hutchinson et al. (2019) observed that crocodylian species reduce speed and eventually lose any asymmetrical gait capacity at only moderate sizes (and probably during ontogeny). Giant (1000–3000+ kg; Table S1) extant and extinct Crocodylia can still walk terrestrially and there has been little study of how they maintain this modest capacity (but see Scheyer et al., 2019). Giant rodents are another captivating case study. Although they never evolved to become as large as elephants (Table S1), extinct >500 kg rodents pushed the limits of what their lineage could do as large tetrapods on land. It remains enigmatic to what extent ancestral ‘phylogenetic baggage’ versus biomechanical constraints have shaped the limits of maximal size or athleticism in lineages of giants (Box 3).

### Conclusions

The paucity of extant giant terrestrial tetrapods challenges us to look into Deep Time, and integrate these inferences with neontological data to formulate a comprehensive body of knowledge for evolutionary biomechanics. But gigantism is rare, and often short-lived in geological time scales. Larger taxa face greater risks of extinction during rapid environmental changes (Bakker, 1980; Janis and Carrano, 1992; Payne et al., 2009). For this reason, Dick and Clemente (2017) considered gigantism ‘maladaptive’, but this risks the orthogenetic or teleological thinking that has long plagued the study of land giants (Cope, 1896; Osborn, 1922). The evolutionary trade-offs with gigantism (e.g. offence/defence, efficient locomotion or resource-domination versus slow reproduction and evolution) will be highly environment-specific, as exemplified by the >170 million years of evolutionary history in giant dinosaurs, which only the freak accident of an extraterrestrial impact ended. Rhinoceroses alone reveal that, even at giant sizes, surprising locomotor performance can persist, and extinct lineages hint that this has evolved repeatedly – the ‘elephant solution’ is not the only solution for land giants. McPhee et al. (2018) described fragmentary remains of a giant early-Jurassic sauropodomorph and speculated, on the basis of its robust forelimb bones, that it adopted more crouched forelimb poses (∼low EMA) relative to giant sauropods with uncontroversially columnar forelimbs (higher EMA). Although the robust bones might have been specializations related to feeding or other behaviours, and need further investigation in a biomechanical context, they remind us that fossil taxa could have deviated from prevalent patterns in extant giants. Perhaps giant bipedal theropods such as *Tyrannosaurus* could achieve brief aerial phases or a bouncing ‘grounded run’ (Gatesy et al., 2009; Sellers et al., 2017). But elephants also reveal that their ‘solution’ is not so simple as merely sedate walking, either. A nuanced approach to the evolutionary biomechanics of land giants is important for unravelling the perplexing mysteries that they continue to pose.

## Supplementary Material

Supplementary information
