# Supplementary material for: The evolutionary biomechanics of locomotor function in giant land animals
Source: J Exp Biol. 2021 Jun 8;224(11):jeb217463. doi: 10.1242/jeb.217463 (PMC8214834; doi:10.1242/jeb.217463)
Supplement: Supplementary information [file jexbio-224-217463-s1.pdf]

Table S1. Dataset for taxa, geological time context, body masses (estimated/typical known) and references. See Fig. 1 for details.

Click here to download Table S1

Table S2. Dataset for maximal running speeds vs. body masses of extant mammals, modified from Dick and Clemente (2017), with notes/references for modifications. See Fig. 5 for details.

Click here to download Table S2

Table S3. Dataset for limb effective mechanical advantages (EMA) vs. body masses of extant mammals and other taxa, modified from Biewener (2005), with notes/references for modifications. See Fig. 3 for details.

Click here to download Table S3

Journal of Experimental Biology • Supplementary information
